# Association entre le paludisme et le faible poids de naissance à Yaoundé, Cameroun

**DOI:** 10.11604/pamj.2019.33.127.18101

**Published:** 2019-06-21

**Authors:** Maurice Ebode Ela, Samuel Nambile Cumber, Rama Djouedjon Dakenyo, Dorine Djuissi Tekam, Patrick Charles Biyong Heumou, Giresse Lowe Marvin, Jerome Ateudjieu, Eva Fomo Tsakoue

**Affiliations:** 1 Faculté de Médecine et Sciences Pharmaceutiques, Université de Dschang, Dschang, Cameroun; 2 School of Health Systems and Public Health, Faculty of Health Sciences, University of Pretoria, Gezina, Pretoria, South Africa; 3 Section for Epidemiology and Social Medecine, Department of Public Health, Institute of Medecine (EPSO) The Sahlgrenska Academy at University of Gothenburg, Gothenburg, Sweden; 4 Faculty of Health Sciences, University of the Free State, Bloemfontein, South Africa

**Keywords:** Faible poids de naissance, association, paludisme, femme enceinte, Yaoundé, Cameroun, Low birth weight, association, malaria, pregnant woman, Yaoundé, Cameroon

## Abstract

**Introduction:**

Le faible poids de naissance (FPN) est un important prédicteur de survie de l’enfant et de son développement ultérieur. De par sa physiopathologie, le paludisme est présumé en être un des facteurs de risques. La présente étude avait pour but de déterminer l’association entre l’accès palustre chez la femme enceinte (FE) et la survenue du FPN (poids < 2500 g).

**Méthodes:**

Il s’est agi d’une enquête analytique de type cas-témoins, basée sur l’administration d’un questionnaire et d’une grille d’observation. Nous avons calculé l’Odds ratio (OR) brut et l’OR ajusté pour déterminer l’association. La régression logistique a été appliquée pour reconnaître les variables qui sont effectivement les déterminants du problème étudié.

**Résultats:**

Cent cinquante-six (156) femmes dont 78 cas et 78 témoins ont participé à la présente étude. La prévalence de FPN était de 12,32% (105/852); 41,02% (64/156) des femmes ont eu un accès palustre durant leurs grossesses et 42,14% de parturientes ont pris leurs trois doses de TPI (traitement préventif intermittent). Nous avons ressorti une association significative entre le paludisme et le FPN avec un OR brute = 3,75 [P = 0,0001 (p < 0,05)] et l’OR ajusté = 2,82 [P = 0,01 (p < 0,05)] a été obtenu en tenant compte des différents facteurs de confusions.

**Conclusion:**

La survenue du paludisme durant la grossesse est un facteur qui augmente le risque de FPN. Les efforts devraient être faits pour améliorer la couverture en TPI et en utilisation de moustiquaire imprégnée d’insecticide à longue durée d’action afin de prévenir le paludisme durant la grossesse.

## Abstract (en)

**Introduction:**

Low birth weight (LBW) is an important predictor of newborn survival and development. Given its pathophysiology, malaria is presumed to be one of the risk factors for low birth weight. This study aims to determine the association between malaria in pregnant women (PW) and LBW (weight < 2500 g).

**Methods:**

We conducted a case-control analytical study based on the administration of a questionnaire and an observation chart. We calculated the crude odds ratio (OR) and the adjusted odds ratio to determine this association. Logistic regression was applied to recognize the variables which act as determinants of the issue under discussion.

**Results:**

This study involved 156 women (78 cases and 78 controls). The prevalence of LBW was 12.32% (105/852); 41.02% (64/156) of women had had malaria during pregnancy and 42.14% of parturients had received three doses of IPT (intermittent preventive treatment). A significant association between malaria and LBW emerged. Crude odds ratio= 3.75 [P = 0.0001 (p < 0.05)] and adjusted OR = 2.82 [P = 0.01 (p < 0.05)] were calculated taking into account the various confusion factors.

**Conclusion:**

Malaria during pregnancy is a factor increasing the risk of LBW. Efforts should be made to improve IPT coverage and the use of long lasting impregnated mosquito nets in order to prevent malaria during pregnancy.

## Introduction

L'UNICEF (2017) [1] défini le faible poids de naissance (FPN) comme un poids de naissance inférieur à 2500g. Chaque année, plus de 20 millions d’enfants ont à la naissance un poids inférieur à 2500g: 10,1% en Amérique, 6,5% en Europe et 14% en Afrique. Il ressort de l'Enquête Démographique de Santé du Cameroun de 2011 (EDS-MICS) [2] que le poids à la naissance des enfants est connu dans 59% des cas. Parmi ces naissances vivantes, 10% avait un faible poids (inférieur à 2500g). Il convient ici de distinguer parmi les nouveau-nés de faible poids de naissance: les enfants hypotrophes (RCIU: retard de croissance intra-utérin) et des enfants prématurés. Les nouveaux-nés ayant un FPN risquent de mourir pendant les premiers mois ou les premières années de leur existence, ces derniers courent plus de risquent d’avoir un système immunitaire déficient, de connaitre des problèmes de santé et de développement, des difficultés d’apprentissage, des déficiences auditives et visuelles, des problèmes respiratoires chroniques tels que l’asthme et les maladies chroniques plus tard au cours de leur vie [3]. Le FPN augmente la morbidité et mortalité infantile, du fait de ses complications multiples à savoir l’hypothermie, l’hypoglycémie, l’asphyxie périnatale, la détresse respiratoire, les troubles hydro électrolytiques, l’hyperbilirubinémie, l’anémie, les troubles nutritionnels, les infections, les troubles neurologiques et les complications ophtalmologiques [4]. Le poids à la naissance est donc un important indicateur de santé du nouveau-né avant et pendant la grossesse; c’est aussi un important prédicteur de la survie de l’enfant et de son développement ultérieur [5].

Parmi les principaux facteurs associés au FPN nous avons: le jeune âge des mères, les antécédents de FPN, le surménage physique des mères, la primiparité, les soins prénataux inadéquats, les risquent infectieux: c’est le cas du paludisme qui contribue à augmenter le taux de risque d’anémie chez les femmes enceintes [6]. Les interventions mise en œuvre au Cameroun durant la deuxième réunion de consensus organisée en janvier 2014 à Yaoundé pour prévenir le paludisme chez les femmes enceintes (FE) sont: l’administration de la Sulfadoxine-Pyrimethamine (SP) aux femmes enceintes pendant les consultations prénatales a été adoptée en raison du nombre important de cas de paludisme chez ces dernières et de ses conséquences néfastes sur la santé de la mère et de l’enfant. Il est recommandé le traitement préventif intermittent (TPI) à toutes les femmes enceintes où les doses doivent être données lors des consultations prénatales régulières dès le deuxième trimestre de la grossesse; la distribution des MILDA (Moustiquaire Imprégnée d’insecticide à Longue Durée d’Action). Nous nous interrogeons: malgré la mise en œuvre de toutes ces interventions, est-ce que les femmes continuent à faire des accès palustres durant la grossesse. Ces accès palustres sont-ils suffisamment graves pour entrainer le FPN chez le nouveau-né?

Environ 52 millions de femmes en Afrique subsaharienne tombent enceintes chaque année et sont exposées au *Plasmodium falciparum*, forme la plus mortelle du paludisme et ayant la plus grande prévalence sur le continent Africain (OMS, 2017) [7]. Dans une étude menée au Sénégal il en ressort une association significative entre le paludisme et la prématurité (OR = 3,91; p = 0,01) [8], une autre étude effectuée au Cameroun présente l’association entre les fièvres présumées palustres et le Retard de Croissance Intra Utérin (RCIU) [9]. Ces études sont limitées par la définition de l’exposition principale qui est le paludisme que dans certaines se définit comme la fièvre présumée palustre et dans d’autres se définit comme étant une Goutte Epaisse (GE) positive. La particularité de notre étude est que nous avons fait une combinaison des deux à savoir fièvre plus GE positive documentés dans le dossier du malade. Nous sommes partis de l’hypothèse de recherche selon laquelle il y’a une forte proportion des cas de FPN attribuable à l’accès palustre au Cameroun. L’objectif était de déterminer l’association entre le paludisme et le FPN et d’identifié les différents facteurs y associés. Nous nous sommes proposés: d’estimer la prévalence de FPN chez les FE, d’évaluer le protocole de prévention du paludisme chez les FE et de déterminer la fréquence du paludisme au cours de la grossesse.

## Méthodes

**Figure 1:**
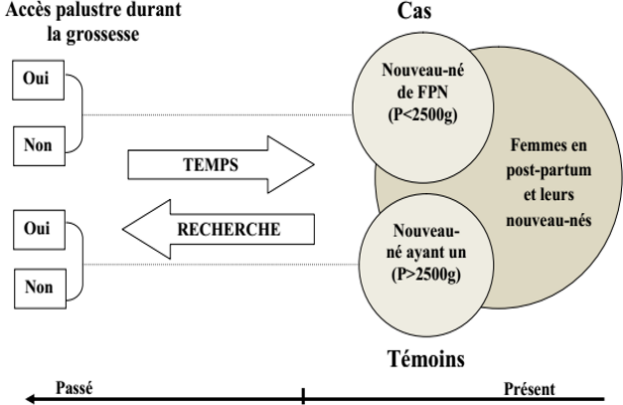
Étude cas-témoins (rétrospective)

Il s’est agi d’une étude analytique de type cas-témoins ou le cas était le nouveau-né présentant un poids < 2500 g et le témoin le nouveau-né ayant un poids > 2500g. L’exposition représente l’accès fébrile plus un examen de la GE positive (preuve de paludisme) durant la grossesse chez la femme enceinte et la non exposition représente l’absence d’un accès fébrile et d’une preuve de paludisme au cours de la grossesse. L’exposition était recherchée dans chaque groupe afin de déterminer l’association entre le paludisme et le FPN (Figure 1). Nous avions pour population source: les femmes en post-partum et leurs nouveaux-nés, et comme population cible les femmes enceintes et nouveaux-nés admis dans les services de maternité et de néonatalogie de l’Hôpital Central de Yaoundé.

### Critères de sélection

Étaient inclus dans notre étude tous les nouveaux-nés vivants et leurs mères.

**Pour les cas:** 1) tout nouveau-né vivant dont le poids de naissance était inférieur à 2500g, admis et ayant séjourné dans les services de maternité et d’unité de néonatalogie pendant la période d’enquête; 2) toute mère ayant donné son consentement pour participer à l’étude.

**Pour les témoins:** 1) tout nouveau-né vivant dont le poids de naissance était supérieur à 2500g, admis et ayant séjourné dans l’unité de néonatalogie et les services de maternité pendant la période d’enquête; 2) toute mère ayant donné son consentement pour participer à l’étude.

### Critères d’exclusion

Étaient exclus de notre étude, les couples mères-enfants ayant des dossiers ne contenant pas des renseignements sur les variables étudiées.

### Stratégies de recrutement de la population d’étude

Premièrement nous recherchions le statut du nouveau-né selon les critères suivants.

**Le poids à la naissance:** nouveau-né ayant un poids à la naissance inférieur à 2500g, ce dernier pouvant être: 1) **prématuré:** nouveau-né né avant 37 SA et pesant au moins 500g; 2) **RCIU ou hypotrophie:** nouveau-né dont le poids à la naissance est inférieur au 10^ème^ percentile (peut-être à terme). Ensuite nous évaluions la disponibilité des différentes variables recherchées dans les dossiers des parturientes afin de pouvoir mesurer l’association entre l’exposition et le FPN durant l’analyse. Comme exposé et non-exposé nous avions: 1) **exposé:** toute femme en post-partum ayant fait au moins un accès palustre durant la gestation; 2) **non-exposé:** toute femme en post-partum n’ayant pas fait d’accès palustre durant la gestation.

### Calcul de la taille d’échantillon

Pour déterminer le nombre d’enfants à enquêter, nous avons procédé suivant le Tableau 1. Nous observons à la table 6d page 45 du sample size determination in health studies [10] que pour OR = 2 et P = 0,40 [2] (le paludisme est à l’origine de 40% du FPN); la taille de l'échantillon doit être de 66 pour chacun des deux groupes. Nous avons utilisé le rapport un cas pour un témoin. Ainsi nous avions obtenu une taille d’échantillon égale à 132 nouveau-nés et leurs mères respectivement dans notre enquête. Dans le but d’améliorer la puissance de notre étude, et de palier aux non répondants, nous avons fait un recrutement exhaustif. Ainsi nous avons recruté 156 nouveau-nés et leurs mères.

**Tableau 1:** Calcul de la taille d’échantillon

Indicateurs	Valeurs
Valeur testée du rapport de côte	1
Probabilité anticipée des « exposés » à donner la « maladie », Probabilité anticipée des « exposés » à ne pas donner la « maladie » (Assimilée aux taux d’exposition globale)	40 %
Rapport de côte anticipé	2
Niveau de signification	5%
Niveau de confiance	95%
Précision relative	50%
Hypothèse alternative	Odds ratio # 1

### Procédure de collecte de données

Le questionnaire adressé aux mères des nouveaux-nés et la grille d’observations des carnets de suivis prénatals étaient pré-testés dans deux maternités autres que celle de l’HCY. Une fois dans la salle de couche, l’entretien entre l’investigateur et la parturiente débutait par la remise d’une notice d’information à la participante. Ensuite, l’enquêteur prenait le soin de lui expliquer cette notice jusqu’à ce qu’elle la comprenne correctement. Par la suite la participante exprimait son accord en remplissant le formulaire de consentement éclairé. Après cela, l’investigateur administrait le questionnaire en face en face, puis remplissait la fiche d’observation à l’aide du carnet de consultations prénatales.

### Procédure d’analyse des données

Les données ont été introduites uniquement par l’investigateur principal dans le logiciel Epi Info 7 pour l’analyse. Lors de cette analyse nous avons tenu compte d’une variable dépendante et de plusieurs variables indépendantes réparties en 03 grands groupes (Caractéristiques sociodémographiques des mères, Suivi de la grossesse Antécédents obstétricaux des mères).

L’Odds-ratio (OR) brut et l’Odds ratio ajusté ainsi que leurs intervalles de confiance à 95% ont été généré pour estimer le sens d’association entre le FPN et l’accès palustre en tenant compte des différents facteurs de confusion. L’analyse proprement dite a été faite selon le modèle unie variée: nous avions calculé les rapports de côte (OR) pour chacune des variables indépendantes avec leur intervalle de confiance (IC) à 95%. Ensuite seuls les variables dont l’IC > 1 étaient entrées dans le modèle de régression logistique multivariée. Dans ce modèle, nous avons observé pour chacune des variables incluses, le rapport de côte ajusté (AOR) à l’ensemble des variables entrées dans le modèle de régression logistique et son intervalle de confiance à 95%. Au terme de cette analyse, une variable ayant un OR ajusté > 1 et un intervalle de confiance supérieur à 1 était considérée comme variable ayant un effet significatif pour la survenue d’un nouveau-né de FPN, indépendamment des autres variables. Les statistiques descriptives usuelles ont été utilisées pour générer les différentes proportions et fréquences. Le test de *Chi-carré* a été appliqué pour l’analyse des tables de contingence et le *test t de student*pour la comparaison des moyennes des variables quantitatives. Les différents indicateurs étaient estimées avec un degré de signifiance alpha (α) = 0,05 sont regroupés dans le Tableau 2.

**Tableau 2:** Formule de calcul de quelques indicateurs pris en compte dans l’étude

Indicateurs	Estimation de l’indicateur
Pourcentage de femmes enceintes dormant sous une MILDA	(Nombre de femmes enceintes utilisant une MILDA /Nombre total de femmes enceintes ayant participé à l’enquête) × 100
Pourcentage de femmes enceintes ayant reçu la SP	(Nombre de femmes enceintes ayant pris leurs 3 doses de SP/Nombre total de femmes enceintes ayant participéà l’enquête) ×100
Pourcentage de femmes enceintes ayant eu un accès palustre durant la grossesse	(Nombre de femmes enceintes ayant fait une fièvre + GE positive+ traitement antipaludéen / Nombre total de femmes enceintes ayant participé à l’enquête) ×100
Pourcentage de nouveau-né de faible poids de naissance (<2500g)	(Nombre total d’enfants nés de FPN/Nombre total d’enfants ayant participé à l’étude) ×100
Odds Ratio Brute	Croisement d’un tableau de contingence contenant : des exposes (fièvre + GE positive), de non exposés, des cas et des témoins. OR= Rapport entre la côte d’être exposé chez les cas et la côte d’être exposé parmi les témoins.
Odds Ratio Ajusté	Ont été inclus dans l’ajustement des variables avec OR>1

### Considération éthique

L’approbation éthique CE N°0090/CRERSHC/ST/2017/ a été obtenue auprès du Comité Régional d’Ethique de la Recherche pour la Santé. Les autorisations administratives N°0087 /AP/MINSANTE/SG/DRSPC et N°086/AR/MINSANTE/SG/DHCY/AAMP, obtenues de la Délégation Régionale de la Santé Publique du Centre et l'autorisation de l'exercice de recherche a été accordée par le Directeur de l'Hôpital Central de Yaoundé. Tous les participants à l'étude ont obtenu un consentement verbal avant l'administration du questionnaire avec l'assurance de respecter la confidentialité.

## Résultats

Au cours de notre période d’étude allant du 04 avril au 25 juin 2017, 852 naissances vivantes ont été enregistrés. Soit 12,32% (105/852) nouveaux-nés de FPN. Dès 105 nouveaux-nés de FPN, nous avons recruté 78/105 (74,29%) nouveaux-nés de FPN (cas) et les 27/105 (25,71%) restant n’ont pas été inclus du fait que leurs dossiers étaient incomplets. Parmi les 747 nouveaux-nés de poids > 2500g, 78/747 (10,44%) ont été sélectionné de manière apparier au cas.

### Caractéristiques des mères

#### Activités principales des parturientes

Le Tableau 3 donne la distribution des mères par profession. On note que les élèves et étudiantes ont été plus représentées au cours de notre étude avec 23/78 (29,48%) chez les cas et 17/78 (21,79%) chez les témoins.

**Tableau 3:** Répartition des parturientes selon la profession chez les cas et témoins

Profession	Cas	(%)	Témoins	(%)
Ménagère	19	24,36	13	16,67
Vendeuse-commerçante	9	11,54	11	14,10
Elève - étudiante	23	29,48	17	21,79
Fonctionnaire	9	11,54	22	28,21
Autres	18	23,08	15	19,23
Total	78	100	78	100

Autres**:** couturière (18) ; coiffeuse (12) ; esthéticienne (3)

### Comparaison des caractéristiques des mères par groupe (cas et témoins)

Le Tableau 4 donne la comparaison des caractéristiques des mères par groupe de cas et témoins. On note que l’adolescence (OR = 4,04 et p value < 0,05), le statut célibataire (OR = 2,12 et p value < 0,05), les primigestes (OR = 2,69 et p value < 0,05), la fièvre (OR = 2,58 et p value < 0,05), douleurs abdominales (OR = 3,4 et p value < 0,05), infection génitales (OR = 2,42 et p value < 0,05) et VIH/SIDA (OR = 3,19 et p value<0,05) ont une association significative avec la survenue de FPN. Nous constatons aussi que le fait d’avoir fait un nombre de CPN > 4 avaient un effet protecteur sur la survenue de FPN avec un OR = 0,24 et une valeur significative (P < 0,05).

**Tableau 4:** Présentation des différentes variables en fonction des cas et témoins

Caractéristiques	Cas	%	Témoins	%	OR	p value
Paludisme	44	56,41	20	25,64	3,75	0,0001
Age (≤ 19 ans)	14	17,95	4	5,12	4,04	0,018
Célibataire	58	74,36	45	57,69	2,12	0,029
Primipare	35	44,87	24	30,77	1,83	0,07
Primigeste	32	41,03	16	20,51	2,69	0,006
0 CPN	4	5,13	1	1,28	4,16	0,206
4 CPN	5	6,41	12	15,39	0,24	0,009
Fièvre	52	66,67	34	43,58	2,58	0,004
Douleurs abdominales	38	48,72	17	21,79	3,4	0,0006
Infection génitales	30	38,46	20	25,64	2,42	0,015
ATCD avortement	22	28,20	15	19,23	1,4	0,356
ATCD de FPN	15	19,23	10	12,82	1,61	0,278
VIH/SIDA	14	17,95	5	6,41	3,19	0,034
HTA	10	12,82	5	6,41	2,14	0,182
Anémie	5	6,41	4	5,12	1,26	0,731

ATCD: Antécédent CPN: Consultation prénatale

### Terme de la grossesse chez les parturientes

**Figure 2:**
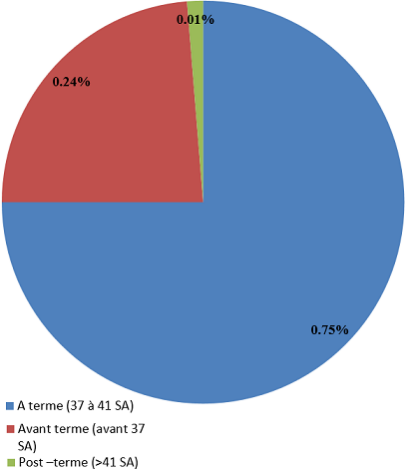
Répartition en fonction du terme de la grossesse

Soixante (75%) (117/156) de nos parturientes étaient à terme au moment de l’accouchement, 23,72% (37/156) étaient avant terme et 1,28% (2/156) étaient en post terme lors de leur accouchement (Figure 2).

### Prévalence de Faible Poids de Naissance

#### Catégories de Faible Poids de Naissance

Parmi les 105 nouveau-nés de FPN enregistrés durant notre période d’étude, nous avons inclus 78/105 (74,29%) nouveaux-nés de faible poids de naissance, répartis ainsi: 37/78(47,44%) prématurés et 41/78(52,56 %) hypotrophes.

#### Distribution des nouveau-nés de FPN par poids

**Figure 3:**
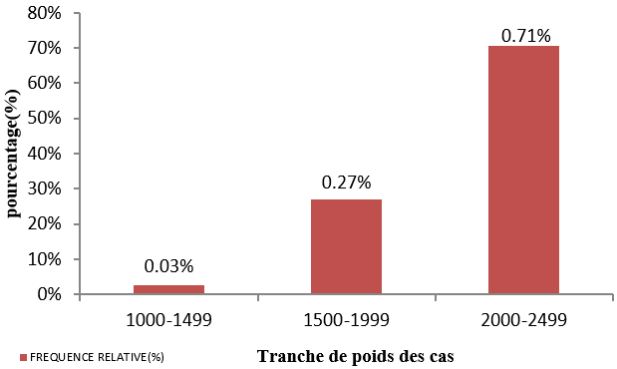
Distribution des cas par poids

La Figure 3 nous illustre les tranches de poids de nouveau-nés à la naissance chez les cas. Ces poids variaient entre 1440g et 2470g. Il en ressort que le poids de naissance moyen est de 2124 ± 252.45g et le poids médian est estimé à 2160g. Le premier quartile est de 1940g. Le troisième quartile est de 2340g et l’espace interquartile va de 1940g à 2340g. La majorité de nouveau-nés de faible poids de naissance ce situe dans la tranche de 2000g à 2499g avec 70,51%.

### Couverture en interventions visant à prévenir le paludisme

#### Couverture en sulfadoxine pyrimethamine

Concernant le nombre de dose de TPI (Traitement Préventif Intermittent), nous avons constaté que 42,14% des parturientes ont pris leurs trois doses de TPI avec une p value non significative (p = 0,24) et un OR = 0,68 montrant l’effet protecteur du TPI sur la survenue de FPN. Ledit résultat peut être apprécier sur le Tableau 5.

**Tableau 5:** Répartition des parturientes en fonction du nombre de dose de TPI

Sulfadoxine-Pyrimethamine	Globalement		cas		témoins		OR	P-Value
	n	%	n	%	n	%		
1 dose	21	15	11	14,10	10	12,82	1,11	0,81
2 doses	58	41,43	32	41,03	26	33,33	1,39	0,32
3 doses	59	42,14	26	33,33	33	42,31	0,68	0,24
4 doses	2	1,43	0	0	2	2,56	0	0,96

#### Couverture sur l’utilisation de MILDA et acide folique

Nous notons des effets protecteurs sur l’utilisation d’une MILDA sur la survenue du FPN avec une significativité avérée (P < 005) (Tableau 6).

**Tableau 6:** Répartition des parturientes en fonction de l'utilisation de MILDA et acide folique

Interventions	Cas	%	Témoins	%	OR	p value
Acide folique et fer	67	91,78	76	97,44	0,29	0,1416
MILDA	56	71,79	74	94,87	0,13	0,0005

### Fréquence du paludisme et association sur la survenue de FPN

De nos parturientes 64/156(41,02%) ont eu au moins une goutte épaisse positive durant leur grossesse avec une association entre le fait d’avoir eu une GE positive et la survenue de FPN avec OR = 3,75 et une valeur P significative (P = 0,0001).

Odds Ratio = (44*58) / (34*20)

**OR (Odds ratio) = 3,75** (ceux-ci voudrait dire que le paludisme augmente de 3,75 fois le risque pour une femme de faire un nouveau-né de faible poids de naissance).

**IC à 95% = 1, 90 - 7, 38**

**P value = 0, 0001** (Statistiquement Significative).

Le paludisme était significativement lié au faible poids de naissance avec un OR_brute_ = 3,75 (P Value = 0,0001).

Après ajustement du paludisme par les potentiels facteurs de confusion tels que: l’adolescence, célibat, primigeste, l’absence de CPN, fièvre, douleurs abdominales, infections génitales, primipare, ATCD d’avortement, ATCD de FPN, anémie, VIH/SIDA, HTA le paludisme reste significativement lié au faible poids de naissance avec un OR_ajusté_ = 2,82 (P Value = 0,01). Le Tableau 7 ci-nous présente la répartition de ces facteurs chez les cas et témoin.

**Tableau 7:** OR ajusté du paludisme avec les facteurs associés au FPN

Caractéristiques	Cas	%	Témoins	%	OR ajusté	p value
**Paludisme**	**44**	**56,41**	**20**	**25,64**	**2,82**	**0,01**
Adolescence	14	17,95	4	5,12	2,66	0,16
Célibataire	58	74,36	45	57,69	1,4	0,42
Primigeste	32	41,03	16	20,51	2,72	0,03
0 CPN	4	5,13	1	1,28	15,60	0,02
Fièvre	52	66,67	34	43,58	1,54	0,29
Douleurs abdominales	38	48,72	17	21,79	4,09	0,0008
Infection génitales	30	38,46	20	25,64	2,26	0,06
Primipare	35	44,87	24	30,77	0,49	0,37
ATCD avortement	22	28,20	15	19,23	2,42	0,10
ATCD FPN	15	19,23	10	12,82	1,55	0,42
HTA	10	12,82	5	6,41	1,37	0,68
VIH/SIDA	14	19,95	5	6,41	1,47	0,59
Anémie	5	6,41	4	5,12	1,51	0,62

## Discussion

La principale limite de notre étude est celui de biais d’information qui révèle du fait que certaines parturientes ne se sont pas bien rappeler sur l’exposition, le déroulement et le suivi de la grossesse ou autres questions jugées embarrassantes pour elles. Par ailleurs, nous avons rencontré quelques difficultés importantes lors du déroulement de cette étude. Notamment l’indisponibilité de certaines informations concernant le suivi de la grossesse et l’insuffisance de notification des prestations durant la grossesse par les prestataires de soins. L’objectif principal de ce travail était la mise en exergue de l’association entre le paludisme et le FPN dans le service de maternité de l’HCY. Pour ce faire, nous nous sommes fixé une hypothèse qu’il y’a une proportion des cas de faible poids de naissance attribuable à l’accès palustre au Cameroun. Les résultats obtenus nous suggèrent des interprétations qui seront développées plus bas au regard des autres travaux et des faits scientifiques à notre portée.

### Estimation de la prévalence de FPN durant la période d’étude

#### Prévalence de FPN

Notre prévalence est supérieure à la moyenne européenne [11] et à ceux trouvés par Nouhoum D au CSR (Centre de Santé la Référence) de la commune V à Bamako [12] qui sont respectivement de 6% et 6,83%. Il est inférieur à la moyenne internationale qui est de 17% [1] et est approximativement égal à celle obtenue par N’Diaye *et al.* (2006) [8]. Les taux africains élevés par rapport aux pays occidentaux pourraient s’expliquer par le fait que la plupart de nos pays africains ont un bas niveau socio-sanitaire ainsi qu’une différence de définition de terme de grossesse.

**Catégories des nouveaux-nés** Concernant la catégorie de FPN, dans notre étude il en ressort 47,44% de prématurés contre 52,56% de cas de RCIU. Cette fréquence élevée de RCIU suggère une grande participation de pathologies de la mère au cours de la grossesse notamment les infections urinaires, l’hypertension artérielle. La proportion de prématuré dans notre étude se rapproche de celle obtenue par Beddeck *et al.* en 2013 en Algérie [6] rapportant une prévalence de 50,9%. Nos résultats vont dans le même sens que ceux obtenus par Nouhoum dans le centre de santé de la commune V de Bamako ayant obtenu 38,65% de prématurés et 61,35% d’hypotrophiques [12].

### Fréquence et évaluation du protocole de prévention du paludisme chez les parturientes

#### Consultations prénatales

Nous notons que 4/78 (5,13%) de nos parturientes n’ont pas fait de CPN chez les cas contre 1/78 (1,28%) chez les témoins et 5/78 (6,41%) parturientes avaient fait plus de quatre CPN chez les cas contre 12/78 (15,39%) chez les témoins. Les facteurs tels que la primiparité, le problème de couverture sanitaire de la région et le bas niveau socio-économique pourraient expliquer ces différentes variations. Nous constatons une forte association entre le fait de n’avoir pas fait de CPN et la survenue de faible poids de naissance avec une valeur P non significative (P > 0,05). Nos résultats concordent avec ceux obtenus par Blondel *et al.* (1993) [13] qui trouva le principe selon lequel la proportion de nouveaux-nés de FPN est toujours significativement plus élevée chez les femmes peu suivies que les autres femmes [14] avec le principe selon lequel le risque de FPN est réduit chez les femmes qui ont suivi des CPN.

### Traitement préventif intermittent/sulfadoxine pyrimethamine (SP)

Nos parturientes (42,14%) ont pris 3 doses de chimioprophylaxie antipalustre pendant leur grossesse avec une OR = 0,68 et une valeur p = 0,24. Cette proportion est légèrement inférieure à celle obtenue dans une étude menée au Mali qui trouva que 72% affirmaient avoir suivi un traitement préventif intermittent complet à la SP au cours de la grossesse [15].

### Utilisation d’une MILDA

Nous constatons que 83,33% de nos parturientes utilisaient une MILDA. Ce taux est comparé à celui obtenu par Famanta [16] dans une étude menée au Mali avec une proportion de 80,07% des parturientes qui utilisaient une moustiquaire imprégnée (MI) cette couverture en utilisation des MILDA pourrait être due à une augmentation de la couverture des MILDA dans les ménages à l’issu de la récente campagne de distribution de moustiquaire dans la région du centre.

### Paludisme et autres facteurs de risques d’intérêts durant la période d’étude*

#### Pathologies associes à la grossesse

Le paludisme a été la pathologie la plus rencontrée au cours de la grossesse dans notre étude; l’accès palustre était significativement lié au FPN avec un OR brute = 3,75 (p < 0,05). Après ajustement du paludisme par les potentiels facteurs de confusion tels que: hypertension artérielle, VIH/SIDA, ATCD de FPN, ATCD d’avortement, anémie, primiparité, adolescence, le statut célibataire, infection génitale, le paludisme reste significativement lié au faible poids de naissance. Cela veut dire qu’une mère qui a fait un accès palustre avait trois fois plus de risque que son nouveau-né soit de faible poids de naissance par rapport à une mère n’ayant pas eu un accès palustre pendant sa grossesse. Nos résultats vont dans le même sens que ceux obtenus par Ndiaye *et al.* [8] qui trouva que le paludisme était significativement associé au faible poids de naissance avec un OR = 3,91 corroborant aussi ceux de Menendez *et al.* [17]. Ceci pourrait s’expliqué par la situation géographique de la région qui se trouve dans la zone de forte endémie palustre et que l’accouchement de FPN serait lié à l’accès palustre et à l’envahissement parasitaire du placenta interférant avec le transfert de l’oxygène et des nutriments. Nous avons obtenu: 29,49% de cas d’infection urinaire et 5,77% de cas d’anémie contrairement à celui trouvé par Mounkoro, [12] qui est de 3,7% de cas d’anémie. Ce taux qui est légèrement élevé dans notre étude pourrait s’expliquer par un niveau socio-économique précaire, l’insuffisance de CPN, et de supplémentation martiale systématique des femmes enceintes.

## Conclusion

La survenue du paludisme au cours de la grossesse est un facteur augmentant le risque de FPN. Ces résultats confirment la faible couverture des différentes interventions visant à prévenir le paludisme chez les femmes enceintes tel que noté dans les Directives National. Il serait donc important et bénéfique d’aménager d’avantage des efforts pour améliorer la couverture visant à prévenir le paludisme durant la grossesse à l’instar du TPI et de l’utilisation des MILDA.

### Etat des connaissances actuelles sur le sujet

- Le paludisme est la première cause de morbidité et de mortalité chez les femmes enceintes au Cameroun;
- L’association de plusieurs mesures prophylactiques améliore la prévention du paludisme;
- Un poids insuffisant à la naissance est une cause majeure de mortalité et de morbidité dans la petite enfance et le FPN est un important indicateur de santé et du suivi de la grossesse.

### Contribution de notre étude à la connaissance

- Apprécier la faible couverture des mesures prophylactique anti-palustre chez les femmes enceintes;
- De connaitre le réel poids du paludisme chez la femme enceinte sur la survenue de FPN et de ressortir l’impact d’un mauvais suivi de grossesse sur la survenue du FPN;
- La chimioprophylaxie avec la SP est, avec la MILDA, les mesures pouvant réduire la survenue de FPN.

## Conflits d’intérêts

Les auteurs ne déclarent aucun conflit d’intérêts.
